# Effect of nalbuphine and morphine as adjuvants to bupivacaine in ultrasound-guided supraclavicular block: a randomized controlled trial with machine learning-based predictive analysis

**DOI:** 10.1186/s12871-025-03537-6

**Published:** 2025-12-28

**Authors:** Alzahraa A. Abbas, Sameh Ghoneim, Mohamed Sharf, Ahmed Farag

**Affiliations:** 1 Department of Anesthesia, Intensive Care and Pain Management, Damietta Faculty of Medicine for Girls, Al-Azhar University, New Damietta, Egypt; 2 Department of Anesthesia, Intensive Care and Pain Management, Faculty of Medicine for Girls, Al-Azhar University, Cairo, Egypt; 3 Department of Anesthesia, Intensive Care and Pain Management, Damietta Faculty of Medicine, Al-Azhar University, New Damietta, Egypt

**Keywords:** Analgesia, Artificial intelligence, Brachial plexus block, Bupivacaine, Machine Learning, Morphine, Nalbuphine, Nerve block, Prediction, Supraclavicular block, Ultrasound

## Abstract

**Background:**

Supraclavicular brachial plexus block is a commonly used regional anesthesia technique for upper extremity surgeries. The addition of adjuvants to local anesthetics enhances the duration and quality of anesthesia. Recently, Machine Learning (ML) has emerged as a tool for predictive modeling in medicine, including pain management. This study investigates the predictive capability of a K-Nearest Neighbor (KNN) ML model for analgesic duration using different drug combinations.

**Methods:**

A prospective randomized controlled trial was conducted on 60 patients scheduled for upper limb surgeries under ultrasound-guided supraclavicular brachial plexus block. Patients were divided into three groups: Control (bupivacaine + saline), Nalbuphine (bupivacaine + nalbuphine), and Morphine (bupivacaine + morphine). The analgesic duration, sensory and motor block onset and duration, and postoperative analgesic use were recorded. A ML model, K-Nearest Neighbor (KNN), was developed to predict analgesic time based on demographic and hemodynamic parameters.

**Results:**

Both nalbuphine and morphine significantly prolonged the duration of analgesia compared to the control group. The KNN model demonstrated a strong correlation (*R* = 0.95) between the observed and predicted analgesic duration, indicating high predictive accuracy.

**Conclusions:**

Nalbuphine and morphine significantly extended the analgesic duration of bupivacaine in ultrasound-guided supraclavicular brachial plexus block. ML models, such as KNN, offer effective tools for predicting analgesic outcomes and can assist anesthesiologists in making informed decisions regarding drug combinations for enhanced patient care.

**Trial registration:**

ClinicalTrials.gov (NCT07008443). Registered in June 2025. Retrospectively registered. ClinicalTrials.gov is a primary registry in the WHO International Clinical Trials Registry Platform (ICTRP) network.

**Supplementary Information:**

The online version contains supplementary material available at 10.1186/s12871-025-03537-6.

## Introduction

The supraclavicular approach to the brachial plexus block has become a widely used technique for providing effective regional anesthesia during upper extremity surgeries [1, 2]. This method is favored for its high success rate and reliability in achieving anesthesia of the entire arm, making it a critical procedure in the field of anesthesia [3]. However, despite its effectiveness, the duration of analgesia provided by the local anesthetic, bupivacaine, often requires augmentation through the addition of adjuvants such as nalbuphine and morphine to prolong pain relief and reduce the need for postoperative analgesics [4].

Recent advances in the field of anesthesia have introduced Machine Learning (ML) as a promising tool for improving decision-making processes. ML refers specifically to algorithms that can learn patterns from data and generate predictions or classifications without being explicitly programmed [5]. In anesthesia and pain management, ML has shown increasing potential—particularly for tasks such as monitoring depth of anesthesia, optimizing opioid dosage, ultrasound-guided nerve blocks, and predicting postoperative pain requirements [6, 7]. However, despite these advancements, a significant gap remains in leveraging ML to predict analgesic duration in regional anesthesia when adjuvants are combined with local anesthetics [8]. A comparison of common ML techniques applied in anesthetic practice is presented in Table 1, summarizing their clinical applications, key strengths, and limitations.

**Table 1:** Summary of related ML applications in anesthesia and pain management

Study/Reference	ML Method	Clinical Application	Strengths	Limitations
[9]	Random Forest	Predicting postoperative pain	Handles nonlinear relationships; robust	Requires feature tuning; can overfit
[10]	Neural Network	Depth of anesthesia monitoring	Good performance on time-series data	Black-box model; limited transparency
[11]	Support Vector Machine	Adverse event classification	Effective with small, high-dimensional data	Sensitive to parameter settings
[12]	Gradient Boosting	Opioid dosage optimization	High accuracy; well-suited for imbalanced data	Prone to overfitting without regularization

This study aims to address the noted gap by employing an ML model—specifically, the K-Nearest Neighbor (KNN) algorithm—to predict the duration of analgesia when nalbuphine or morphine is added to bupivacaine in ultrasound-guided supraclavicular brachial plexus blocks. We hypothesize that ML-based models can accurately predict analgesic duration and contribute to optimizing anesthesia management, offering anesthesiologists a supportive tool to enhance patient care. The primary aim of this study is to evaluate the effectiveness of nalbuphine and morphine as adjuvants to bupivacaine in prolonging the analgesic duration during ultrasound-guided supraclavicular brachial plexus block. Additionally, this research seeks to assess the feasibility of using AI to predict analgesic outcomes based on clinical and demographic parameters [13, 14].

## Methods

This double-blind, prospective, randomized clinical trial was conducted at Al-Zahraa and Damietta University Hospitals, following ethical approval (No. 731, dated 21 December 2022) granted by the Anesthesia Department’s Research Ethics Committee, Al-Azhar Faculty of Medicine for Girls (AFMG) at Al-Azhar University, Cairo, Egypt. Written informed consent was obtained from all patients prior to participation.

### Participants

A total of 60 adult patients aged 21 to 60 years, scheduled for elective surgeries of the upper limb distal to the elbow, were included. Eligible participants were classified as ASA physical status I or II. Patients were excluded if they had drug allergies, coagulation disorders, significant comorbidities, or refused participation.

### Randomization and group allocation

Participants were randomly allocated into three groups using a computer-generated list, with assignments sealed in opaque envelopes. Group C (control) received 25 ml of 0.5% bupivacaine plus 5 ml saline, Group N received 25 ml of 0.5% bupivacaine plus nalbuphine (50 µg/kg), and Group M received the same volume with morphine (50 µg/kg). Blinding was maintained for patients, investigators, and care providers.

### Intervention

Each patient underwent an ultrasound-guided supraclavicular brachial plexus block while positioned supine, with the head rotated away from the surgical site. A linear ultrasound probe visualized the plexus and subclavian artery. Using an in-plane technique, a 22G needle delivered 30 ml of the study mixture in a single injection.

### Outcome measures

The primary endpoint was the duration of analgesia, measured from block administration to first postoperative analgesic request. Secondary outcomes included onset/duration of sensory and motor block, paracetamol consumption, patient satisfaction (VAS), and incidence of complications such as pneumothorax or nerve injury.

### Ultrasound-guided supraclavicular brachial plexus block anesthesia technique

Preoperative assessment included clinical examination and laboratory tests (CBC, LFTs, RFTs, coagulation profile), plus ECG and chest X-ray. Standard intraoperative monitoring included NIBP, ECG, and SpO2. An IV line was established in the contralateral limb with Ringer’s lactate (10–20 ml/kg), and supplemental oxygen was delivered at 5 L/min. The block was performed under aseptic conditions with real-time ultrasound guidance using a lateral-to-medial in-plane needle approach. The target area was the plexus around the subclavian artery above the first rib.

### Postoperative evaluation

Block characteristics were assessed every 5 min for 30 min. Sensory block was scored on a 3-point scale, while motor function was graded using a modified Bromage scale. Pain was rated using a 10-cm visual analog scale (VAS) at 2, 6, 12, and 24 h. Paracetamol was administered if VAS ≥ 4, and sedation was assessed with the Ramsay scale. Hemodynamic parameters were recorded every 15 min. Complications such as vascular puncture, Horner syndrome, or respiratory events were monitored and recorded.

### Sample size and analysis

The sample size was calculated in accordance with CONSORT 2010 guidelines based on the primary outcome, duration of analgesia. Calculation was derived from prior data reported by Vengadessane et al. (2020), which indicated that 60 participants (20 per group) would provide 80% power to detect a clinically meaningful difference at a 5% significance level (α = 0.05). The estimated effect size and standard deviation were adopted from this reference, and an additional allowance for potential attrition was incorporated. Statistical analyses were conducted using SPSS version 24 (IBM, USA). Continuous variables were expressed as mean ± standard deviation (SD) or median (range), while categorical data were presented as frequencies and percentages. Data normality was tested using the Shapiro–Wilk test. Depending on data distribution, one-way ANOVA or Kruskal–Wallis tests were applied for continuous variables, and Chi-square tests for categorical variables. When ANOVA revealed significance, Bonferroni-adjusted post-hoc pairwise comparisons were performed to control for multiple testing. A *p* < 0.05 was considered statistically significant.

### K-neighbor regressor (KNR)

The k-Nearest Neighbors (k-NN) algorithm is a widely used supervised machine learning (ML) technique applicable to both classification and regression tasks [15]. In this study, we employed the regression variant of k-NN, commonly referred to as the k-NN regressor (KNR), to predict analgesic duration. The k-NN algorithm is non-parametric and instance-based, making no assumptions about the underlying data distribution and showing robustness to outliers. Its simplicity and interpretability make it suitable for small structured clinical datasets. During training, the algorithm retains the dataset and applies distance-based similarity to estimate outcomes for new data points based on the values of their ‘k’ nearest neighbors. Importantly, k-NN is not a clustering algorithm, as clustering is an unsupervised learning method, whereas k-NN operates under supervised learning [16]. Considering $\:S=\left\{\left({x}_{i},{y}_{i}\:\right);\:\mathrm{i}\:=\:\mathrm{1,2},\:\dots\:\:.\mathrm{n}\right\}$ to be a training set containing n observations for a regression problem. Where $\:{x}_{i}=({x}_{i1}{,x}_{i2}$,…….,$\:\:{,x}_{im})$ is the i-th instance denoted by m features with its response $\:{y}_{i}.$ When new tested data $\:\left({x}_{t},{y}_{t}\right)$, is acquired, it’s required to know how close each testing point to each training point in S by calculating the distance metrics (d) which can be estimated. Then, the distance d is sorted by its value to the closest i-th instance which called the k-nearest neighbor with output $\:{y}_{i}$ (x). Lastly, the final predicted output $\:{y}_{av}$is the average of the outcomes of its k nearest neighbors which can be estimated as follow:

1 $$\:y_{av}=\:\frac1{\mathrm{k}}\sum_{i=1}^k\:{\mathrm{y}}_\mathrm{i}\left(\mathrm{x}\right)$$

Where *k* represents the number of nearest neighbors used for prediction, $\:{y}_{i}\left(x\right)$denotes the output values of the *k* nearest neighbors, and $\:{y}_{av}$refers to the predicted (average) response value for the test instance.

In KNR algorithm, two issues are still challengeable for researchers, the choice of the K-value and the suitable distance metrics parameters [17]. These two parameters must therefore be investigated iteratively in order to get the best KNR’s performance.

The KNR algorithm was selected in this study due to its suitability for small and structured datasets, such as the one used in this study [18]. As a non-parametric, instance-based learning method, KNR requires no assumptions about the underlying data distribution, making it robust in diverse clinical datasets. Its intuitive mechanism—predicting outcomes based on the similarity to nearest neighbors—also allows for explainability, which is crucial in medical decision support [19]. Given the relatively limited sample size (*n* = 60), KNR offered a practical and interpretable approach for building a predictive model of analgesic duration.

## Research methodology

**Fig. 1:**
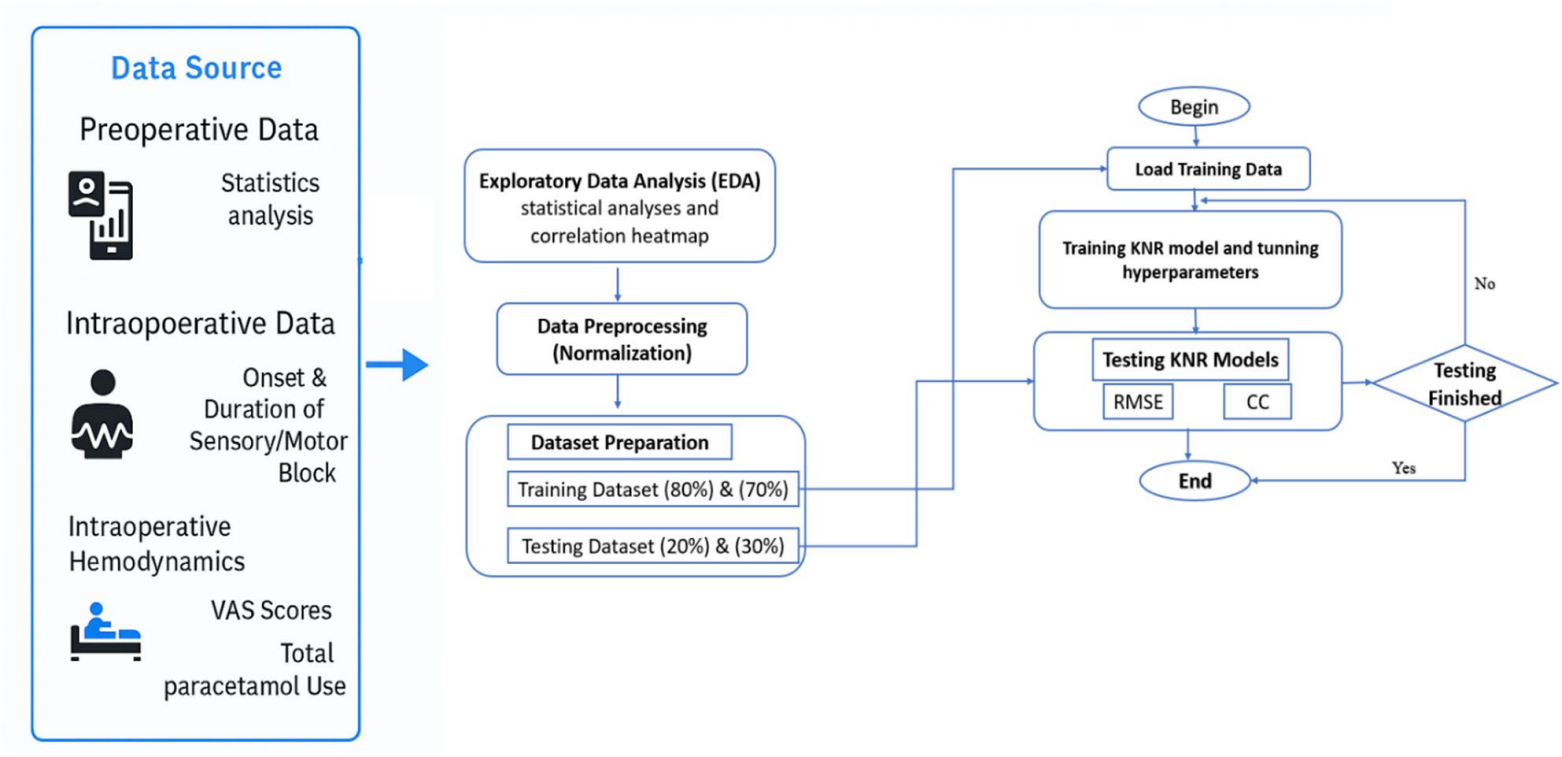
Structured methodology for AI predictive model development

The proposed framework flowchart for forecasting analgesic time is shown in Fig. 1, and the processes of the suggested methodology are shown below:

### Data source

Preoperative, intraoperative and postoperative condition of a patient at different time points are collected and stored in excel sheet.

### Dataset exploration

An exploratory statistical data analysis is performed for searching extensively on variables (demographic, hemodynamics and analgesic time) correlations and system complexity using and statistical analyses and correlation heatmap.

### Data preprocessing

Data preprocessing was performed in accordance with TRIPOD-AI recommendations to ensure data quality, comparability, and reproducibility before model training. The dataset was first examined for completeness, and no missing or outlier values requiring imputation were detected. Because ML algorithms are sensitive to the scale of input variables, standardization was required to avoid magnitude conflicts among predictors with different measurement units (e.g., weight in kilograms versus length in centimeters). Therefore, feature normalization was applied to rescale all variables to a uniform range. Specifically, the Min–Max normalization method was used to scale each variable within the interval (0, 1) according to Eq. (2):

2 $$\:{\mathrm{X}}_{\mathrm{n}}=\:\frac{\:{\mathrm{X}}_{\mathrm{i}}-{\mathrm{X}}_{\mathrm{m}\mathrm{i}\mathrm{n}}}{{\mathrm{X}}_{\mathrm{m}\mathrm{a}\mathrm{x}}-{\mathrm{X}}_{\mathrm{m}\mathrm{i}\mathrm{n}}}$$

Where $\:{\mathrm{X}}_{\mathrm{n}},{\mathrm{X}}_{\mathrm{i}},{\mathrm{X}}_{\mathrm{m}\mathrm{a}\mathrm{x}},{\:\mathrm{a}\mathrm{n}\mathrm{d}\:\mathrm{X}}_{\mathrm{m}\mathrm{i}\mathrm{n}\:}$ are the normalized, original, maximum and minimum values for all parameters respectively.

### Developing KNR model

ML predictive analysis was structured in accordance with the TRIPOD-AI checklist. Predictors included demographic, physiological, and drug-combination variables. The outcome variable was the duration of analgesia (in hours).

kNN regression model was developed using the scikit-learn library (v1.4). The optimal number of neighbors (*k = 3*) and the Euclidean distance metric were selected through grid search and five-fold cross-validation. Feature scaling was applied before training. The data were split into 80% training and 20% testing subsets using a fixed random seed (42) to ensure reproducibility.

### Proposed model evaluation

Model performance was evaluated using the mean absolute error (MAE), root mean square error (RMSE), and coefficient of determination (R²). Calibration between predicted and observed values was plotted and visually inspected. Confidence intervals (95%) were computed by bootstrapping. The utilized evaluation indicators can be computed as follows:

3 $$\:\mathrm{R}\mathrm{o}\mathrm{o}\mathrm{t}\:\mathrm{M}\mathrm{e}\mathrm{a}\mathrm{n}\:\mathrm{S}\mathrm{q}\mathrm{u}\mathrm{a}\mathrm{r}\mathrm{e}\:\mathrm{E}\mathrm{r}\mathrm{o}\mathrm{o}\mathrm{r}\:\left(\mathrm{R}\mathrm{M}\mathrm{S}\mathrm{E}\right)=\:\sqrt{\frac{1}{\mathrm{n}}\:\sum\:_{\mathrm{i}=1}^{\mathrm{n}}{({\mathrm{P}}_{\mathrm{i}}-{\mathrm{M}}_{\mathrm{i}})}^{2}}$$

4 $$\:\mathrm{c}\mathrm{o}\mathrm{r}\mathrm{r}\mathrm{e}\mathrm{l}\mathrm{a}\mathrm{t}\mathrm{i}\mathrm{o}\mathrm{n}\:\mathrm{c}\mathrm{o}\mathrm{e}\mathrm{f}\mathrm{f}\mathrm{i}\mathrm{c}\mathrm{i}\mathrm{e}\mathrm{n}\mathrm{t}\:\left(\mathrm{C}\mathrm{C}\right)=\frac{\sum\:_{\mathrm{i}=1}^{\mathrm{n}}\left({\mathrm{P}}_{\mathrm{i}}-\stackrel{-}{\mathrm{P}}\right)\:\left({\mathrm{M}}_{\mathrm{i}}-\stackrel{-}{\mathrm{M}}\right)}{\sqrt{\sum\:_{\mathrm{i}=1}^{\mathrm{n}}{({\mathrm{P}}_{\mathrm{i}}-\stackrel{-}{\mathrm{M}})}^{2}\:\sum\:_{\mathrm{i}=1}^{\mathrm{n}}{({\mathrm{M}}_{\mathrm{i}}-\stackrel{-}{\mathrm{M}})}^{2}}\:\:}$$

Where ($\:{M}_{i}$), ($\:{P}_{i})$ are the measured and predicted values, *n* is the number of datasets, ($\:\stackrel{-}{M})$, ($\:\stackrel{-}{O})$ are the mean values of measured and predictions, respectively.

## Results

### Statistical analysis

#### Demographic data

There was no statistically significant difference (*P* > 0.05) between the three groups as regards age, weight, height, sex and ASA physical status (Table 2).

**Table 2:** Statistical Criteria of Demographic characteristics used in the three groups of the study

Criteria	Variables	Mean ± SD			*P*
		Group C	Group M	Group N	
Demographic characteristics	Age (years)	34.05 ± 7.93	35.4 ± 8.24	37.7 ± 9.69	0.510
	Gender(male/female)	8/12	9/11	9/11	0.885
	Height (Cm)	162.15 ± 5.89	165.55 ± 6.67	162.30 ± 6.39	0.160
	Weight (kg)	71.89 ± 17.66	72.4 ± 9.85	71.05 ± 10.85	0.623
	ASA (I/II)	16/4	17/3	15/5	0.592

One-way ANOVA was used for unpaired data. Chi-square test was used for paired data (sex and ASA grade)

*P* value was considered statistically significant when < 0.05

Data were represented as mean ± SD or number

*Group C* Control group, *Group N* Nalbuphine group, *Group M* Morphine group, *ASA* American society of anesthesiologists

#### Onset and duration of sensory and motor block and analgesic characters

There were no significant differences in the times of onset of both sensory and motor blocks between the three groups (*P* > 0.05). The analgesic duration was highly significantly longer in N and M groups when compared with the control group. Also, N group and M group significantly longer (*P* < 0.05) regard duration of sensory and motor blocks and time of first analgesia followed by C group. Total rescue analgesia was significantly lower (*P* < 0.05) in N group and M group followed by C group (Table 3).

**Table 3:** Statistical criteria of sensory, motor blocks and analgesic behavior of the drugs used in the three groups of the study

Variables	Mean ± SD			*P*
	Group C	Group M	Group *N*	
Onset of sensory block (min)	10.45 ± 0.53	10.43 ± 0.79	10.54 ± 0.63	0.70
Onset of motor block (min)	20.64 ± 0.65	20.66 ± 0.89	20.51 ± 0.91	0.634
Duration of sensory block (h)	7.62 ± 0.81	14.62 ± 0.48	14.35 ± 0.70	0.000
Duration of motor block (h)	6.80 ± 0.76	13.30 ± 0.59	13.20 ± 0.75	0.000
Duration of analgesia (h)	8.15 ± 0.72	15.55 ± 0.58	15.30 ± 0.61	0.000
Total consumption of paracetamol (g)	2.40 ± 0.5	0.65 ± 0.81	0.75 ± 0.71	0.000

Data were represented as mean ± SD. Group C, control group; group Morphine group; group N, nalbuphine group. One-way analysis of variance was used. *P* < 0.05, statistically significant

#### Pain assessment using visual analogue scale (VAS)

**Fig. 2:**
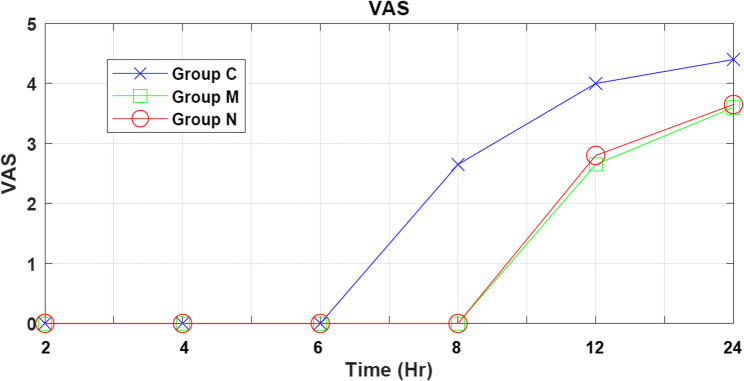
Pain assessment using visual analogue scale (VAS)

VAS was matched until 6th hour as it started to raise especially in control group which was significantly higher (*P* < 0.05) than other groups. These results are summarized in Table 4 and illustrated in Fig. 2.

**Table 4:** Postoperative visual analogue scale (VAS) scores at specified time points across study groups

Variables	Mean ± SD			*P*
	Group C	Group M	Group *N*	
VAS at 2 h	0 ± 0	0 ± 0	0 ± 0	*P* < 0.001
VAS at 4 h	0 ± 0	0 ± 0	0 ± 0	*P* < 0.001
VAS at 6 h	0 ± 0	0 ± 0	0 ± 0	*P* < 0.001
VAS at 8 h	2.65 ± 1.34	0 ± 0	0 ± 0	*P* < 0.001
VAS at 12 h	4.00 ± 0.00	2.65 ± 0.93	2.80 ± 0.76	*P* < 0.001
VAS at 24 h	4.40 ± 0.50	3.60 ± 0.99	3.65 ± 0.87	*P* < 0.001

#### Hemodynamic variables

**Fig. 3:**
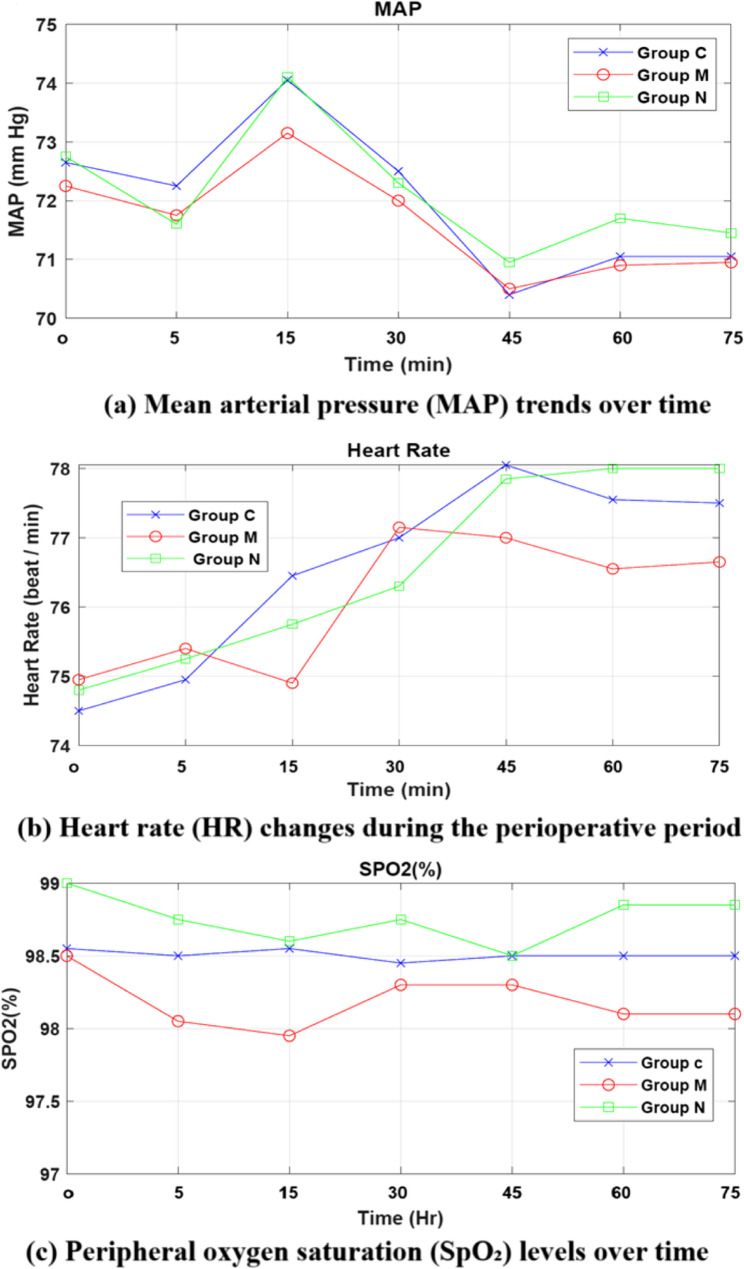
Hemodynamic variables distribution among groups

The trends of hemodynamics parameters (MAP, HR, and SPo_2_) are depicted in Fig. 3. As shown in this figure, no significant difference was found among groups in terms of the hemodynamics parameters for all patients. Also, the obtained statistical analysis results presented in the following tables confirmed these findings where there is no statistically significant difference between the three groups (*P* > 0.05).

##### Respiratory rate (RR)

As regards Respiratory rate (RR), there was no statistically significant difference between the three groups (*P* > 0.05) (Table 5).

**Table 5:** RR at different times during operation among groups

Variables	Mean ± SD			*P*
	Group C	Group M	Group *N*	
RR at 0 min	12.25 ± 0.44	12.25 ± 0.44	12.20 ± 0.41	0.357
RR at 5 min	12.25 ± 0.44	12.25 ± 0.44	12.20 ± 0.41	0.357
RR at 15 min	12.25 ± 0.44	12.25 ± 0.44	12.20 ± 0.41	0.357
RR at 30 min	12.25 ± 0.44	12.25 ± 0.44	12.20 ± 0.41	0.357
RR at 45 min	12.25 ± 0.44	12.25 ± 0.44	12.20 ± 0.41	0.357
RR at 60 min	12.25 ± 0.44	12.25 ± 0.44	12.20 ± 0.41	0.357
RR at 75 min	12.25 ± 0.44	12.25 ± 0.44	12.20 ± 0.41	0.357

##### Ramsay sedation scale

As regards sedation score, there was no statistically significant difference between the three groups (*P* > 0.05) (Table 6).

**Table 6:** Sedation score among groups

Variables	Mean ± SD			*P*
	Group C	Group M	Group *N*	
Sedation Score	2.00 ± 0.45	2.15 ± 0.48	2.10 ± 0.44	0.244

##### Complications

As regards the complications (e.g., pneumothorax, Horner’s syndrome or local anesthetics systemic toxicity), none of the patients in both groups had experienced any side effect or complication either of the anesthetic technique or of the used drugs.

### KNR model development

#### Exploratory data analysis (EDA)

**Fig. 4:**
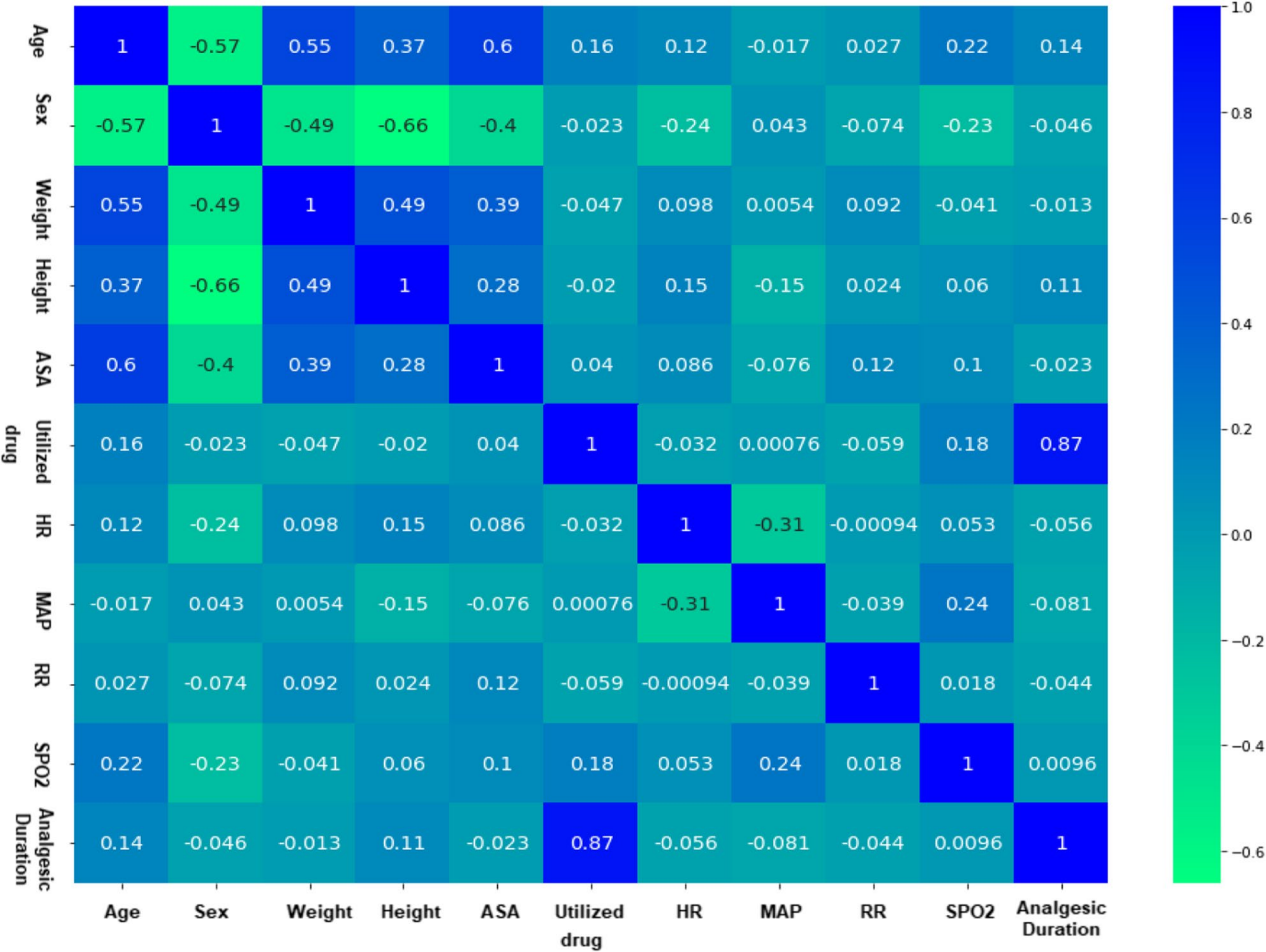
Heatmap matrix of demographic and hemodynamics variables with Analgesic Duration

To gain maximum insight into the collected data set, an EDA is performed to take a bird’s eye view of the data and tries to make some sense of it. For this purpose, a correlation heatmap matrix is executed and automatized by using Python scripting including Seaborn and Matplotlib powerful visualization libraries to describe visually the relation between the different variables as outlined in Fig. 4. The key insights from the conducted EDA can be concluded as: (1) there is no strong correlation between any demographic and hemodynamics parameters with analgesic duration which indicate that predicting analgesic duration is a highly complicated process, and (2) demographic and hemodynamics parameters variables are also interrelated in a complex way as shown by the weak interrelationships. These findings highlight the complexity of the dataset, justifying the use of machine learning specifically the k-NN regression model as a flexible and data-driven alternative to traditional statistical approaches.

### Developing KNR model for predicting analgesic duration

The KNR algorithm was employed to estimate analgesic duration using patient-specific demographic and hemodynamic variables. All analyses were performed in Python 3.11 using the open-source libraries pandas, numpy, and scikit-learn (v1.4). To ensure optimal model performance, a sensitivity analysis was conducted to fine-tune key hyperparameters and identify the most effective combination of settings. The KNR model was executed across 100 iterations, each with varying configurations, as summarized in Table 7. The highest predictive accuracy was achieved using the standardized Euclidean (Seuclidean) distance metric and a neighborhood size of three, yielding correlation coefficients (CC) of 0.96 and 0.95 for the training and test datasets, respectively.

**Fig. 5:**
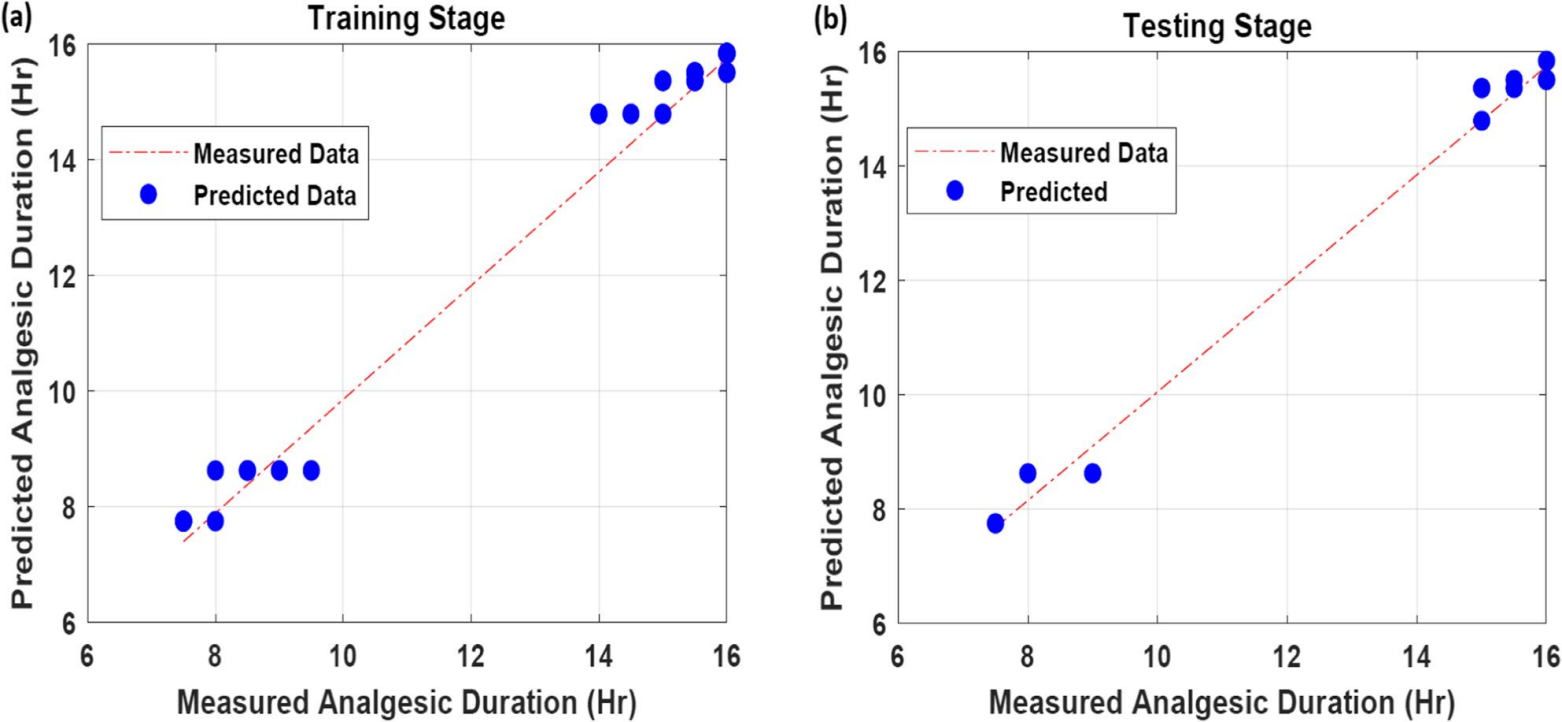
Measured and predicted analgesic duration in training and testing stages using KNN

To evaluate the advantage of using KNR over simpler methods, a comparison was made with a standard linear regression model using the same training and test data. The linear regression model achieved a lower test CC of 0.82 and a higher RMSE = 1.2, indicating that it was less effective at capturing the complex relationships between patient features and analgesic duration. This comparison supports the suitability of the KNR algorithm for this type of clinical prediction task. As illustrated in Fig. 5, the scatter plot reveals a strong correlation between the actual and predicted analgesic durations. Likewise, the series plot demonstrates consistent trends, highlighting the KNR model’s accuracy and reliability in predicting analgesic outcomes with high precision.

**Table 7:** Statistical measures for the KNR algorithm

Model	Hyperparameter	Hyperparameters		Metrics			
		Investigated	Optimum	Training		Testing	
				RMSE	CC	RMSE	CC
KNR	Distance metricss	Euclidean, Seuclidean, Cityblock, cosine	Seuclidean	0.69	0.96	0.73	0.95
	Number of Neighbors (K)	(2–6)	3				
	Data Division Percentage (Training/Testing)	(80/20) & (70/30)	80/20				

## Discussion

### Clinical efficacy of Nalbuphine and morphine

The supraclavicular brachial plexus block is a well-established alternative to general anesthesia for upper limb surgeries. It avoids airway instrumentation, reduces hospital stay and costs, and ensures effective muscle relaxation. The ultrasound-guided approach enhances nerve localization, reduces the required anesthetic volume, and minimizes the risk of intravascular injection or tissue trauma [20].

Effective postoperative analgesia must satisfy safety, efficacy, and practicality. While single-injection local anesthetics provide limited pain relief, adjunctive strategies such as continuous infusions or pharmacologic adjuvants can significantly prolong analgesia. Opioids, dexamethasone, magnesium sulfate, and midazolam have been explored in this context [21].

In this study, nalbuphine and morphine were evaluated as adjuvants to bupivacaine. Both significantly extended the duration of sensory and motor blocks, reduced postoperative analgesic requirements, and resulted in lower VAS scores [22, 23]. These results align with prior findings by Vengadessane et al., [24] and Waly et al. [25]. Our data confirm that even at lower doses, nalbuphine is effective in enhancing block characteristics without delaying onset.

### Machine learning for analgesic prediction

As a secondary objective, we explored the utility of ML, specifically a KNN regression model, to predict analgesic duration based on demographic and physiological parameters. Exploratory data analysis revealed weak correlations among traditional variables, reinforcing the need for a predictive tool. The KNN model showed a strong correlation between predicted and actual outcomes, suggesting the feasibility of using ML in anesthesia planning. However, this application remains exploratory and needs validation with larger, multicenter datasets.

### Study limitations

Several limitations should be noted. The sample size was relatively small (*n* = 60), which may restrict generalizability. The clinical trial was retrospectively registered due to procedural delays. The ML component was developed using single-center data, and its external applicability is not yet established. Furthermore, the model was not compared against other algorithms beyond linear regression, warranting broader benchmarking. Additionally, this study adheres to TRIPOD-AI recommendations, emphasizing transparent reporting of data sources, model development, and validation procedures. However, external validation was not performed, representing a limitation requiring multicenter datasets.

### Future directions

AI may play a transformative role in anesthetic care, particularly in ultrasound-guided block planning, opioid titration, and risk prediction. Future work should focus on real-time integration of ML tools in clinical workflows and prospective validation of predictive models.

## Conclusion

The current study showed that adding nalbuphine or morphine to bupivacaine in US-guided SCBPB extends the duration of analgesia by 8 h over the time of bupivacaine alone and reduces postoperative analgesic requirements, resulting in significantly lower VAS scores throughout the study period. On the other hand, the study confirms that ML technology has the potential to successfully develop efficient decision support systems for estimating analgesic duration based on drug combination.

## Supplementary Information

Supplementary Material 1.

## Data Availability

The data that support the findings of this study are not openly available due to reasons of sensitivity and are available from the corresponding author upon reasonable request.

## Abbreviations

US-guided SCBPB: Ultrasound-guided supraclavicular brachial plexus block
KNN: K-nearest neighbors
KNR: K-nearest neighbors regressor
ML: Machine Learning
VAS: Visual analog scale
ASA: American Society of Anesthesiologists
MAP: Mean arterial pressure
RR: Respiratory rate
